# Special Issue “Feature Review Papers in Organic Synthesis”

**DOI:** 10.3390/molecules27030825

**Published:** 2022-01-26

**Authors:** Rafael Chinchilla

**Affiliations:** Department of Organic Chemistry, Faculty of Sciences and Institute of Organic Synthesis (ISO), University of Alicante, Apdo. 99, 03080 Alicante, Spain; chinchilla@ua.es; Tel.: +34-965903548

Organic synthesis allows scientists to construct and modify organic compounds using a continuously growing arsenal of reactions and methodologies. Consequently, new or improved procedures are reported daily for preparing a wide variety of valuable compounds belonging to the most different fields. This Special Issue of *Molecules* presents contributions reviewing modern synthetic procedures employed in several research areas.

The preparation of products suitable as starting materials for the synthesis of compounds of interest is always a critical point in organic synthesis. The contribution of Verochkina et al. illustrates this point [1], reviewing the synthesis of structurally α-functionalized α,β-unsaturated aldehydes, an important class of compounds widely used in fine organic synthesis, biology, pharmacology, chemical industry, and agriculture. 

The generation of heterocyclic systems is also an important topic in organic synthesis, as they appear in many biologically active compounds. Zhilitskaya et al. [2] present a representative review on this topic, which shows recent synthetic trends in the chemistry of biologically active 2-amino and 2-mercapto substituted benzothiazoles, from conventional multistep processes to one-pot, atom-economy procedures performed using green chemistry principles. In addition, the review of Zhang et al. [3] is another interesting example of heterocyclic chemistry, showing contributions in the design of N-acyl homoserine lactone (AHL) analogues. AHLs are small signalling molecules used by many Gram-negative bacteria, analogues and mimics of AHLs being biologically relevant targets. 

The use of visible light as an energy source in the presence of a photocatalyst is currently a fast-progressing area in organic synthesis, as the experimental setup is more accessible than the one employed in traditional photochemical reactions, and fewer side-reactions usually occur. Concerning this topic, the review of Torregrosa-Chinillach et al. [4] presents recent developments achieved in the aerobic oxidative dehydrogenation of C–N and C–O bonds, leading to C=N and C=O bonds, respectively, using metal-free homogeneous or recyclable heterogeneous photocatalysts.

The development of procedures leading to biomass valorization is emerging as a strong trend due to awareness of the need for sustainable development in reusing waste and biomass. The review of Endot et al. [5] shows the recent advances in the transformation of biobased 5-hydroxymethylfurfural (HMF) into the potential liquid fuel 2,5-dimethylfuran (DMF).

The use of asymmetric synthetic methodologies plays a crucial role in preparing bioactive or other interesting compounds. Developments in this fast-moving area are constant, and the contribution of Maestro et al. is a good example [6]. Thus, this review focuses on the diastereoselective and enantioselective preparation of tetrasubstituted α-aminophosphonic acid derivatives, which are biologically active molecules due to their structural similarity with natural α-amino acids. Related to ensuring the validity of asymmetric methodologies in organic synthesis is the contribution of Han et al. [7], which highlights the necessity of conducting specific tests to evaluate the magnitude of the self-disproportionation of enantiomers (SDE) to ensure the veracity of reported enantiomeric excesses. This SDE always occurs to some extent when an enantioenriched mixture is subjected to physicochemical processes, thus leading to erroneous reporting of the real enantiomeric excess of the sample.

The total synthesis of natural products is represented by the review of Sim et al. [8], which shows a series of syntheses of naturally occurring pladienolides or analogues. These compounds are naturally occurring spliceosome modulators that exhibit interesting structural features and are potential anti-cancer agents. In addition, the review by Borrego-Muñoz et al. [9] illustrates the use of organic synthesis for the preparation of agrochemical agents, presenting a compendium of the recent synthetic methodologies employed for the preparation of antifungal compounds against *Fusorium oxysporium* species. These phytopathogens cause vascular wilt and substantial economic losses in agriculture.

Finally, the contribution by Radha et al. [10] shows strategies, methods and levels of detection involved in fluorescence-based immunoassays to detect troponin I, which is the most specific biomarker for cardiac injury.

This Special Issue illustrates how organic synthesis has confronted the preparation of compounds useful for diverse applications. However, despite all the impressive developments obtained, much work must be carried out to develop more efficient synthetic methodologies applicable to more substrates. There is no doubt that many new procedures in organic synthesis will continue to appear in the future.
